# Identification of a nucleotide metabolism-related signature to predict prognosis and guide patient care in hepatocellular carcinoma

**DOI:** 10.3389/fgene.2022.1089291

**Published:** 2023-01-04

**Authors:** Yu Li, Chunyan Wu, Yingnan Ge, Xi Chen, Li Zhu, Ling Chu, Jia Wang, Meiling Yan, Hao Deng

**Affiliations:** ^1^ Department of Gastroenterology, Suzhou Hospital of Integrated Traditional Chinese and Western Medicine, Suzhou, China; ^2^ Department of General Surgery, Zhangjiagang TCM Hospital Affiliated to Nanjing University of Chinese Medicine, Zhangjiagang, China; ^3^ Department of Hepatopancreatobiliary Surgery, Shenzhen Second People’s Hospital, The First Affiliated Hospital of Shenzhen University, Shenzhen, China; ^4^ Shantou University Medical College, Shantou, China

**Keywords:** hepatocellular carcinoma, prognosis, signature, nucleotide metabolism, treatment

## Abstract

**Background:** Hepatocellular carcinoma is a highly malignant tumor with significant heterogeneity. Metabolic reprogramming plays an essential role in the progression of hepatocellular carcinoma. Among them, nucleotide metabolism needs further investigation.

**Methods:** Based on the bioinformatics approach, eleven prognosis-related nucleotide metabolism genes of hepatocellular carcinoma were screened in this study. Based on the Lasso-Cox regression method, we finally identified a prognostic model containing six genes and calculated the risk score for each patient. In addition, a nomogram was constructed on the basis of pathological stage and risk score.

**Results:** Patients with high-risk score had worse prognosis than those with low-risk. The predictive efficiency of the model was efficient in both the TCGA dataset and the ICGC dataset. The risk score is an independent prognostic factor that can be used to screen chemotherapy drugs. In addition, the risk score can be useful in guiding patient care at an early stage.

**Conclusion:** Nucleotide metabolism-related prognostic model can more accurately predict the prognosis of patients with hepatocellular carcinoma. As a novel prediction model, it is expected to help clinical staff to provide targeted treatment and nursing to patients.

## Introduction

Primary liver malignancy is a highly prevalent disease globally. Hepatocellular carcinoma (HCC) is the most common subtype of primary liver malignancy, often accompanied by cirrhosis and chronic hepatitis (Forner et al., 2018; Llovet et al., 2022). Despite significant advances in the diagnosis and treatment of liver cancer, long-term survival remains poor (Laface et al., 2022). Because of the hidden origin of liver cancer, many patients are diagnosed at a late stage (Singal et al., 2020). In addition, the prognostic status of patients with hepatocellular carcinoma is also directly affected by the heterogeneity of their tumor microenvironment (Ma et al., 2019). Notably, China accounts for nearly half of all new liver cancer cases and deaths globally each year, placing a heavy burden on society and the healthcare system (Shi et al., 2021). Therefore, it is very important to judge the prognosis of HCC patients early. Compared to traditional prognostic models, incorporating mRNA expression at the gene level shows promise in predicting survival in HCC patients (Fu and Song, 2021). Further, by constructing a novel prognostic model, we can guide the risk stratification of hepatocellular carcinoma patients and provide targeted care.

Metabolic reprogramming is one of the main features of tumors (Sun et al., 2022). There are many differences between the metabolic patterns of tumor cells and normal cells. Metabolic reprogramming of cancer can influence tumorigenesis and progression (Zhang et al., 2021). The common substance metabolism includes nucleotide metabolism, lipid metabolism, amino acid metabolism, and glucose metabolism (Zhao et al., 2020; Brunner and Finley, 2021). Among them, nucleotide metabolism, as an important substance metabolism mode, remains worthy to be explored in hepatocellular carcinoma. Studies have shown that dysregulation of nucleotide metabolism can promote tumor growth and is associated with tumor escape (Stine et al., 2022; Wu et al., 2022). Targeting nucleotide metabolism provides new ideas for the therapy of malignant tumors.

In this study we mined six potential molecular markers based on nucleotide metabolism related genes using bioinformatic approach. Based on the expression of these six genes we constructed an accurate prediction model, which can be applied to determine the prognosis of patients with hepatocellular carcinoma and guide chemotherapy. In addition, psychological support and emotional regulation for high-risk group can be a way to improve the quality of patients’ survival.

## Materials and methods

### Processing of data

Clinical information and gene expression profile information of liver hepatocellular carcinoma (LIHC) patients were obtained from the TCGA database (Count format and FPKM format). After data normalization (Log2 (TPM+1)) and removal of patients with missing clinical information and survival time less than 30 days, we included the TCGA database as the training set. The hepatocellular carcinoma data from the ICGC database were extracted as an independent validation set. The expression profile data of the ICGC database were normalized, while the patient information data were processed in the same way as the training set.

### Differential expression analysis and construction of prognostic model

“DEseq2” package was used to identify differentially expressed genes (DEGs) in TCGA-LIHC (Log2|FC|>1, FDR<0.05) (Love et al., 2014). Genes related to nucleotide metabolism were obtained from the MSigDB database (http://www.gsea-msigdb.org/) (REACTOME_METABOLISM_OF_NUCLEOTIDES). Venn plot was used to obtain differentially expressed nucleotide metabolism-related genes. Univariate COX analysis was used to identify prognosis-associated genes. On the basis of the R package “glmnet” we performed Lasso-Cox analysis (Wang et al., 2019). Finally, we obtained the risk score [nucleotide metabolism-related score (NMRS)] for each patient with hepatocellular carcinoma: NMRS = [Coef (gene 1) * Exp (gene 1)] + [Coef (gene 2) * Exp (gene 2)] + ...... + [Coef (gene i) * Exp (gene i)] (Huo et al., 2020). R packages “survminer” and “survival” was used to perform Kaplan-Meier analysis. Furthermore, time-dependent ROC analysis was performed using R package “timeROC.” Based on the R package “rms” (Balachandran et al., 2015), we constructed the nomogram and the calibration curve was used to check the accuracy.

### Enrichment analysis

The “limma” package was used to identify DEGs between high and low NMRS groups (Log2|FC|>1, FDR<0.05) (Ritchie et al., 2015). R package “GSVA” was used to calculate the enrichment score of gene sets in each sample. Based on the R package “clusterProfiler,” Gene Ontology (GO) and Kyoto Encyclopedia of Genes and Genomes (KEGG) enrichment analysis were performed for DEGs between high and low NMRS groups (Wu et al., 2021).

### Chemotherapy drug prediction

The R package “pRRophetic” was used to calculate the minimum drug inhibition concentrations (IC_50_) for the high and low NMRS groups (Geeleher et al., 2014). Using this algorithm we identified the sensitive chemotherapeutic drugs in the high NMRS group.

### Statistical analysis

RStudio (v 4.1.3) was used to perform the data analysis. Differences between categorical variables were tested by chi-square test, and continuous variables by *t*-test or paired samples *t*-test, with *p*-values <0.05 being statistically significant.

## Results

### Identification of 11 nucleotide metabolism-related prognostic genes in hepatocellular carcinoma

**FIGURE 1:**
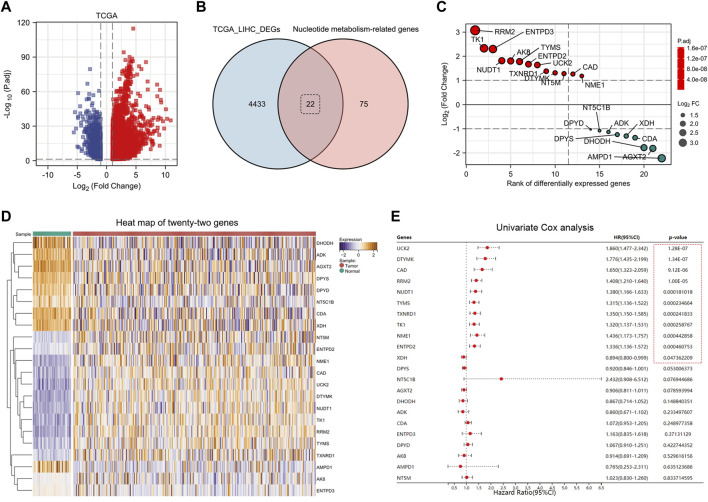
Identification of eleven nucleotide metabolism genes associated with prognosis in hepatocellular carcinoma. **(A)** DEGs in TCGA-LIHC database were identified using DESeq2 algorithm (FDR <0.05, Log2|FC|>1). **(B)** Twenty-two DEGs related to nucleotide metabolism were screened by Venn diagram. **(C)** Fold changes of the twenty-two DEGs. **(D)** Heat map showed the expression differences of twenty-two genes in paracancerous *versus* tumor tissues (after normalization). **(E)** Univariate Cox analysis screened eleven prognosis-related nucleotide metabolism genes.

Nucleotide metabolism-related genes play a huge role in tumor progression. First, we downloaded the patients’ clinical data and genes expression data from the TCGA-LIHC database. Using the R package “Deseq2,” we identified 4,455 DEGs (Figure 1A) (Log2|FC|>1, FDR<0.5). Taking the intersection of DEGs and nucleotide metabolism-related genes, we obtained 22 genes, 13 of which were up-regulated and 9 were down-regulated (Figures 1B,C). By plotting the heat map, we can significantly observe that these 22 nucleotide metabolism-related genes have differential expression in the TCGA database (Figure 1D). To further determine the impact of these genes on the prognosis of LIHC patients, we applied univariate Cox analysis, and finally we identified 11 prognosis-related genes (Figure 1E). They were: UCK2, DTYMK, CAD, RRM2, NUDT1, TYMS, TXNRD1, TK1, NME1, ENTPD2, and XDH.

### A prognostic model with six genes was constructed based on lasso-cox regression analysis

**FIGURE 2:**
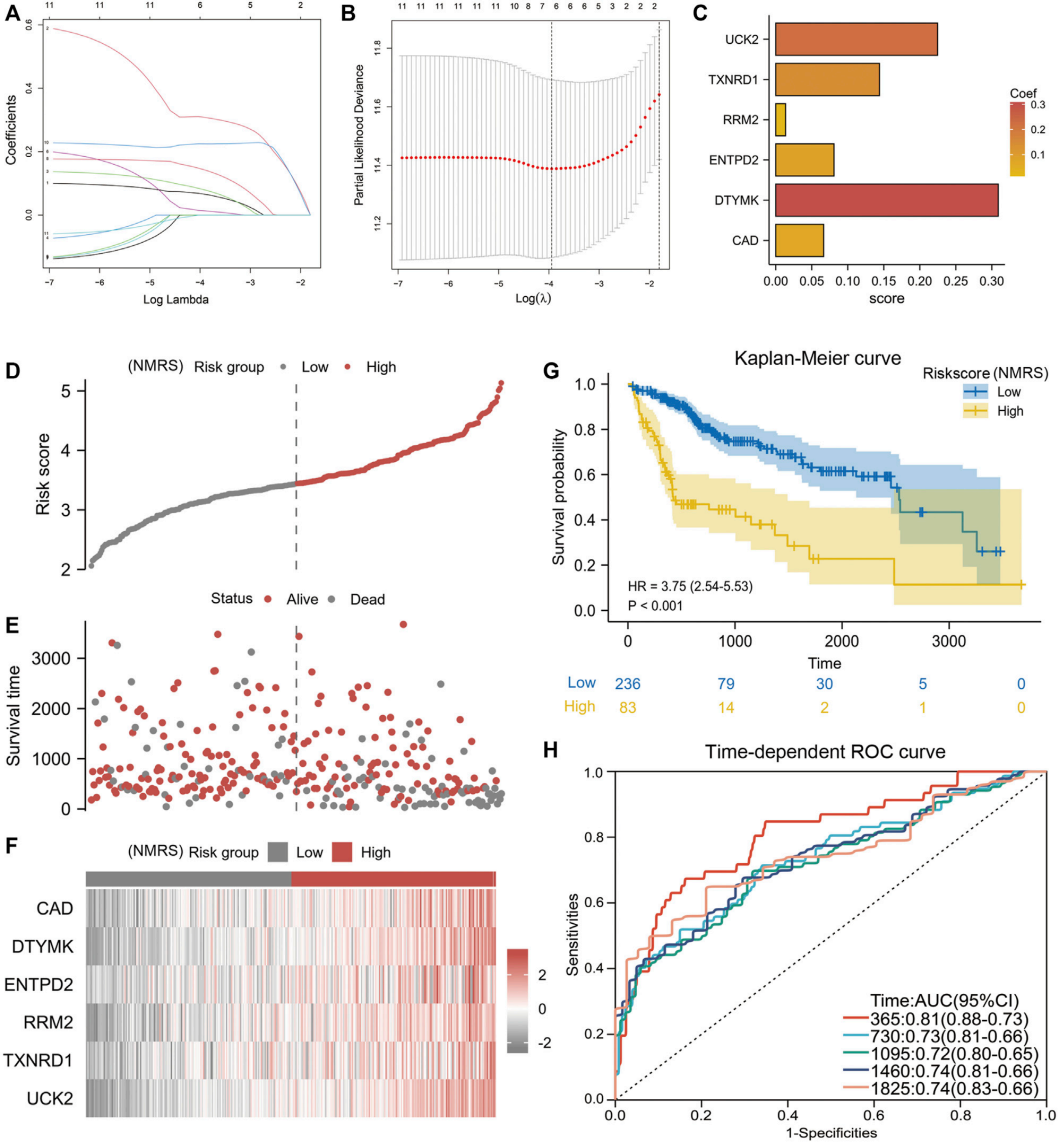
Construction of NMRS. **(A)** Lasso-Cox regression analysis of eleven nucleotide metabolism-related genes. **(B)** Ten-fold cross-validation. **(C)** Six model genes with non-zero coefficients were obtained after screening. **(D)** NMRS were calculated for each hepatocellular carcinoma patient based on the formula, and patients were divided into high and low NMRS groups by median. **(E)** Correlation of NMRS with survival status. **(F)** Differences in the expression of six biomarker genes between the high and low NMRS groups. **(G)** Prognostic differences between patients in the high and low NMRS groups. **(H)** Time-dependent ROC curves to validate the predictive accuracy of the prognostic model.

**FIGURE 3:**
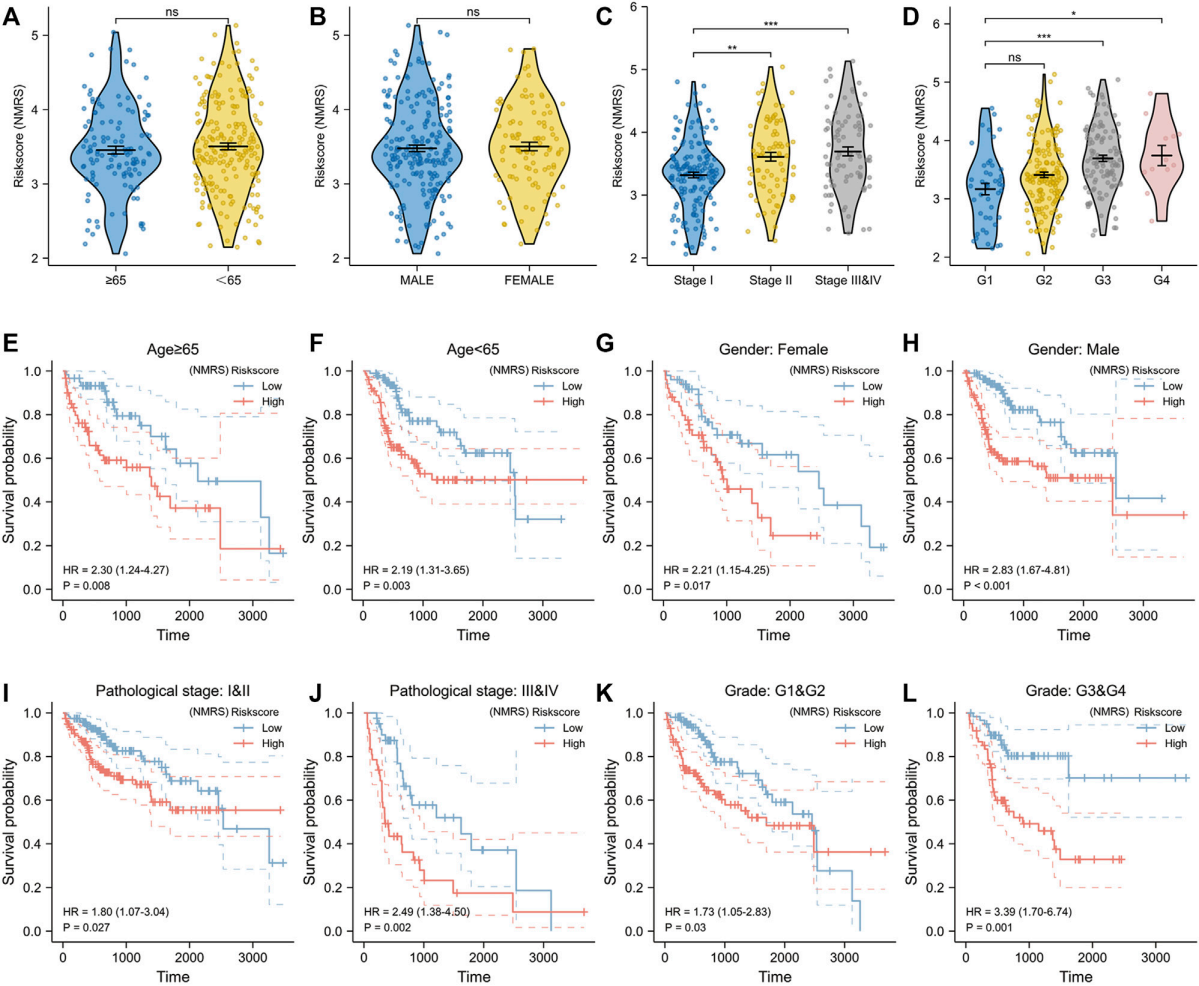
Correlation of NMRS with clinical factors. **(A)** There was no statistical difference in NMRS between hepatocellular carcinoma patients with age≥65 and those with age<65. **(B)** There was no statistical difference in NMRS between male patients and female patients. **(C)** Patients with pathologic stage II or pathologic stage III and IV had higher NMRS compared with patients with pathologic stage **(I) (D)** Patients with Grade G3 & G4 had a higher NMRS. **(E,F)** Kaplan-Meier curves for hepatocellular carcinoma patients aged≥65 and those aged<65. **(G,H)** Kaplan-Meier curves of male patients and female patients. **(I,J)** Kaplan-Meier curves of patients with different pathological stages. **(K,L)** Kaplan-Meier curves of patients with different Grades.

Lasso algorithm can be used to obtain a refined prognostic model by constructing a penalty function. Eleven prognostic genes obtained from the univariate Cox analysis were included in the lasso-cox analysis, and after ten-fold cross-validation, we finally obtained six potential biomarkers with non-zero coefficients (Figures 2A,B). They were UCK2, TXNRD1, RRM2, EMTPD2, DTYMK, and CAD (Figure 2C). Based on the coefficients of these 6 genes, we can calculate the NMRS for each LIHC patient: NMRS = 0.0664731174655317*(exp CAD) + 0.308842373214825*(exp DTYMK) + 0.0808399336082155*(exp ENTPD2) + 0.0136277477123085*(exp RRM2) + 0.143976088249642*(exp TXNRD1) + 0.224626978297361*(exp UCK2). On the basis of the median score, we divided the LIHC patients in the TCGA database into high and low NMRS groups (Figure 2D). The high NMRS group had more deaths, and in addition, six potential markers had significantly higher expression levels in the high NMRS group (Figures 2E,F). After Kaplan-Meier prognostic analysis, we found that patients with high NMRS had significantly shorter survival times (Figure 2G) (HR = 3.75, *p* < 0.001). The time-dependent ROC curves further indicated that the prognostic model we constructed has high predictive accuracy, which is important for us to make early prognostic judgments and enhance management and follow-up of patients (Figure 2H). To further investigate the correlation between NMRS and clinical factors, we performed expression and prognosis analysis. The results showed that the NMRS of patients in the high and low NMRS groups were not statistically significant in terms of age and gender. However, there was a higher NMRS in patients with pathological stages III and IV and Grade 3 and 4 (Figures 3A–D). In addition, the results of the prognostic analysis showed that the high NMRS patients had a worse survival time across clinical factors (Figures 3E–L).

### Potential differences in molecular function between high and low nucleotide metabolism-related score patients

**FIGURE 4:**
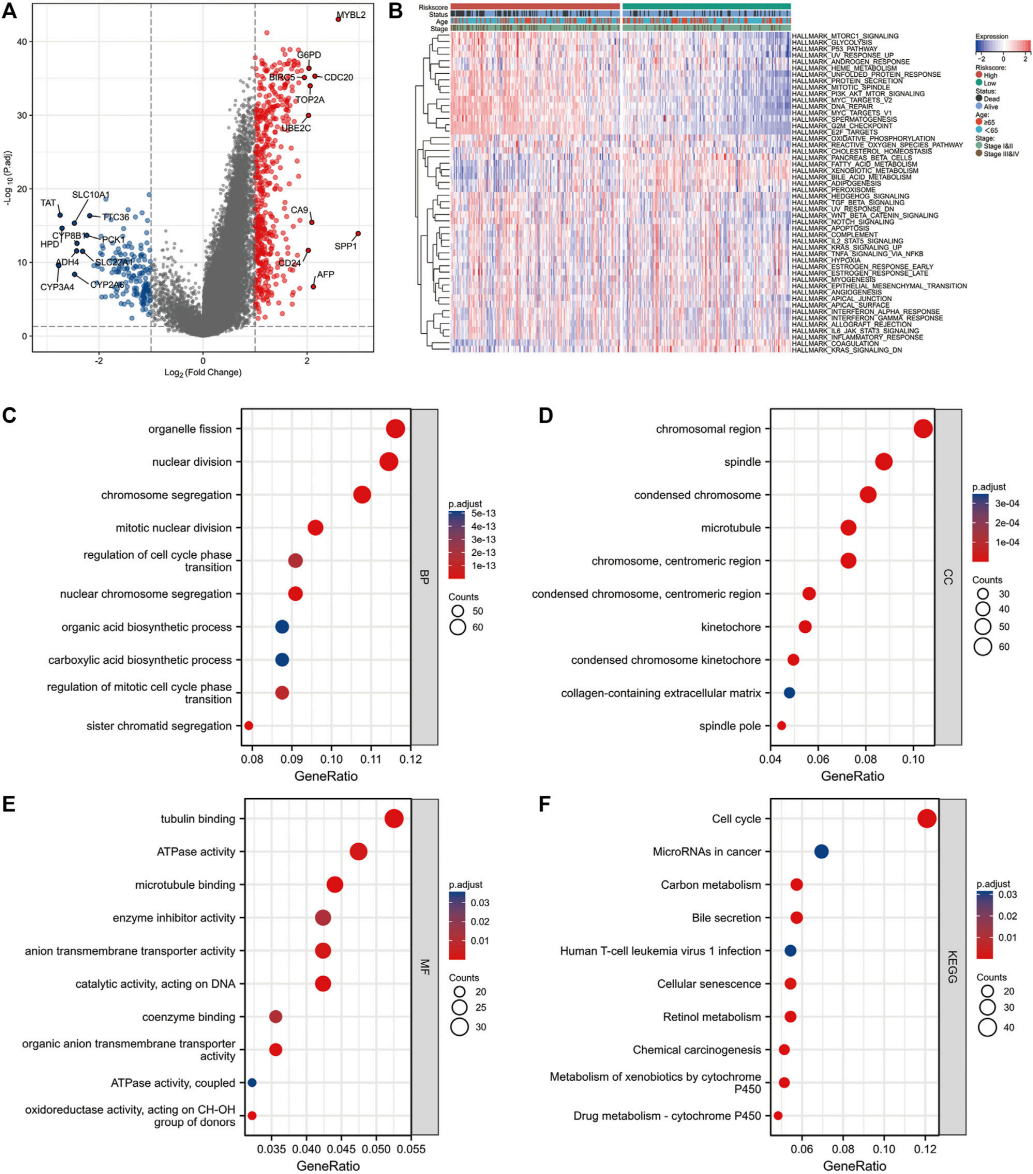
Differences in molecular mechanisms between patients in high and low NMRS groups. **(A)** The “limma” package was used to identify DEGs in high and low NMRS groups, with up-regulated genes in red and down-regulated genes in blue. **(B)** GSVA enrichment analysis was applied to identify differences in activity between patients in high and low NMRS groups on the Hallmark gene sets. **(C–E)** GO enrichment analysis of DEGs. **(F)** KEGG enrichment analysis of DEGs.

R package “limma” was used to perform differential expression analysis between the high and low NMRS groups (Figure 4A). Blue dots represent down-regulated genes and red dots are up-regulated genes (Log2|FC|>1, FDR<0.5). After extracting these DEGs, we performed GO and KEGG enrichment analysis. Figure 4C demonstrated the enriched top10 biological processes; Figure 4D presented the enriched top10 molecular compositions; Figure 4E showed the enriched top10 molecular functions. The results of KEGG showed that the differential genes of this model were mainly enriched in the gene sets of cell cycle, senescence, and so on (Figure 4F). In addition, we performed GSVA enrichment analysis for patients in the high and low NMRS groups (Figure 4B). Based on the 50 Hallmark gene sets, we identified that patients in the high NMRS group had higher enrichment scores in PI3K-AKT-MTOR signaling pathway, GLYCOLYSIS, E2F_TARGETS, MYC_TARGETS, suggesting that patients in the high NMRS group had higher activation in pathways related to tumor progression.

### Independent external dataset to validate the prediction efficiency of the signature

**FIGURE 5:**
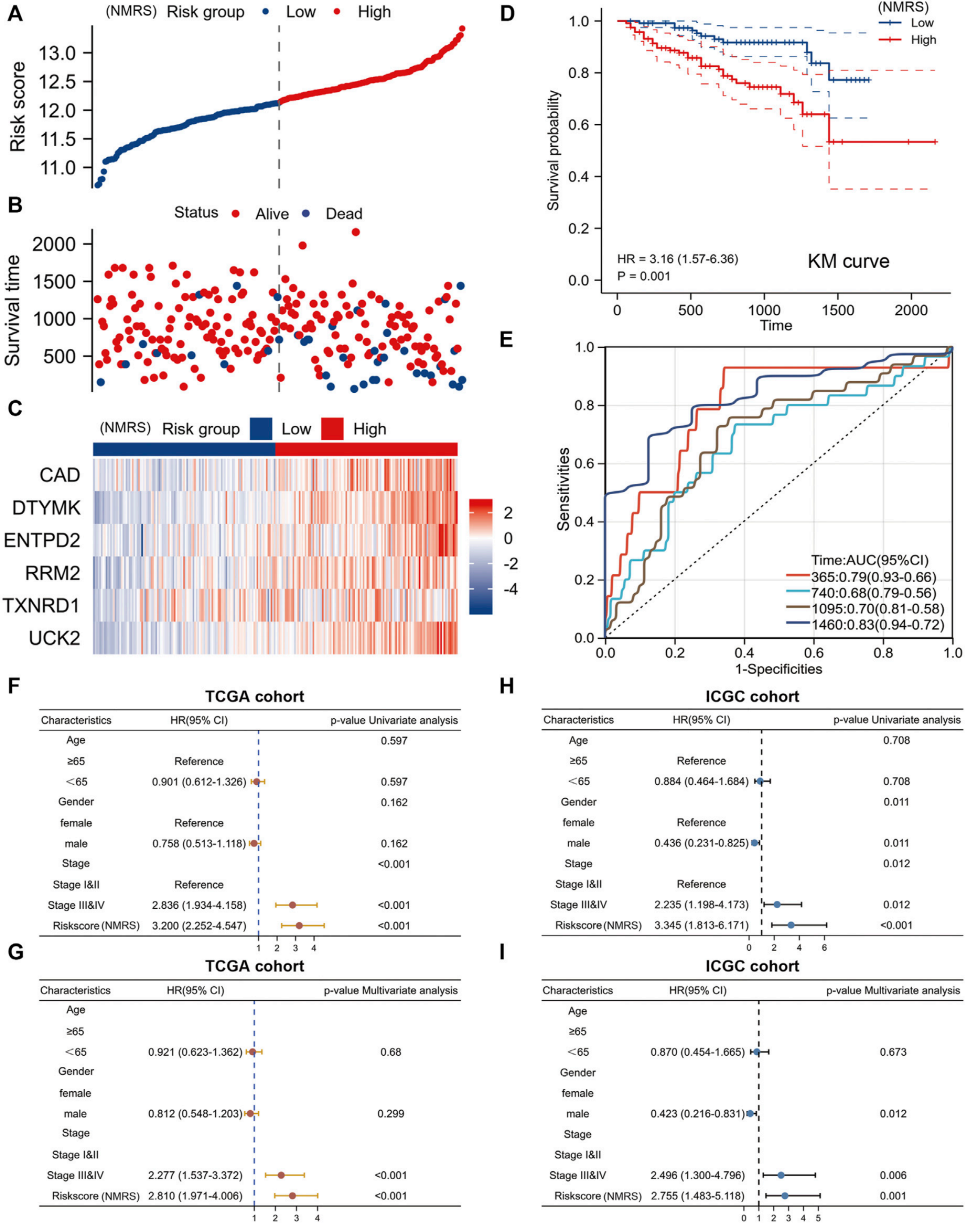
External validation set to verify model efficiency. **(A)** NMRS of patients in the ICGC dataset are calculated based on formula. **(B)** The high NMRS group in the ICGC dataset has more dead patients. **(C)** Differences in expression of six genes in the ICGC database between high and low NMRS groups. **(D)** Prognostic differences between high and low NMRS groups in the ICGC database. **(E)** Time-dependent ROC curve of ICGC database. **(F,G)** Univariate Cox and multivariate Cox analyses of NMRS in the TCGA database. **(H,I)** Univariate Cox and multivariate Cox analysis of NMRS in the ICGC database.

On the basis of the formula described above, we counted the NMRS of each patient in the ICGC database. The ICGC database can be divided into high and low NMRS groups according to the median score (Figure 5A). More patients died in the high NMRS group, and all six genes involved in the construction of the model had higher expression in the high NMRS group (Figures 5B,C). Kaplan-Meier analysis showed that the high NMRS group in the ICGC database had a worse prognosis than the low NMRS group (Figure 5D). The time-dependent ROC curves also showed high accuracy (Figure 5E). In addition, we performed univariate and multivariate Cox analyses of NMRS and clinical factors. In the TCGA database, pathological stage and NMRS were independent prognostic factors (Figures 5F,G). In the ICGC database, gender, pathological stage and NMRS were independent prognostic factors (Figures 5H,I). In conclusion, NMRS as a clinical parameter can be a good adjunct to pathological stage for early determination of patient prognosis.

### Immune checkpoint expression analysis and chemotherapy drug prediction

**FIGURE 6:**
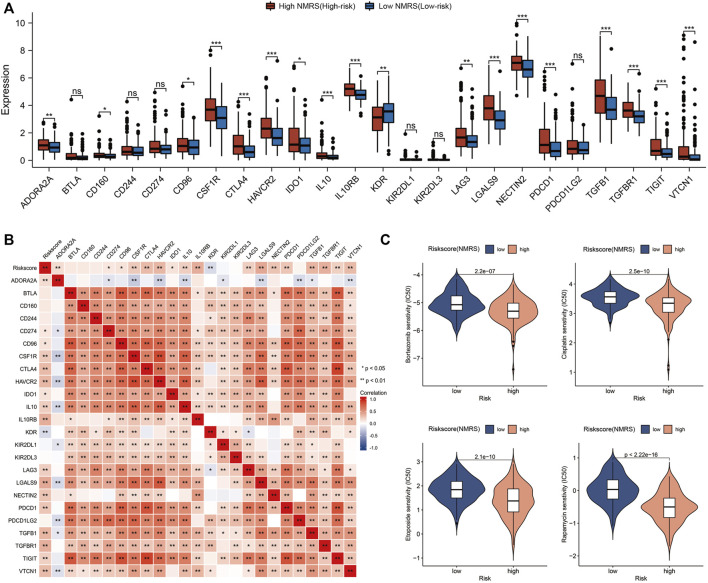
Immune checkpoint analysis and chemotherapy drug prediction. **(A)** Differences in the expression of immune co-inhibitory molecules between high patients and low NMRS patients. **(B)** Correlation analysis of NMRS with immune co-inhibitory molecules. **(C)** Screening of sensitive chemotherapeutic agents in the high NMRS group.

High expression of immunosuppressive molecules correlates with an immunosuppressive microenvironment, which ultimately leads to activation of tumor escape and associates with poor prognosis. Therefore, we extracted the expression profile data of 24 immune co-inhibitory molecules and compared the differences between high and low NMRS groups (Figure 6A). The results indicated that the high NMRS group had higher expression of many common immune co-suppressor molecules such as CTLA4, PDCD1, and TIGIT. The results of correlation analysis also suggested that NMRS was positively correlated with most immune co-inhibitory molecules (Figure 6B). In addition, we also screened four chemotherapy-sensitive drugs for high NMRS group. They were: Bortezomib, Cisplatin, Etoposide, and Rapamycin (Figure 6C).

### Combining pathological stage and nucleotide metabolism-related score to construct a nomogram

**FIGURE 7:**
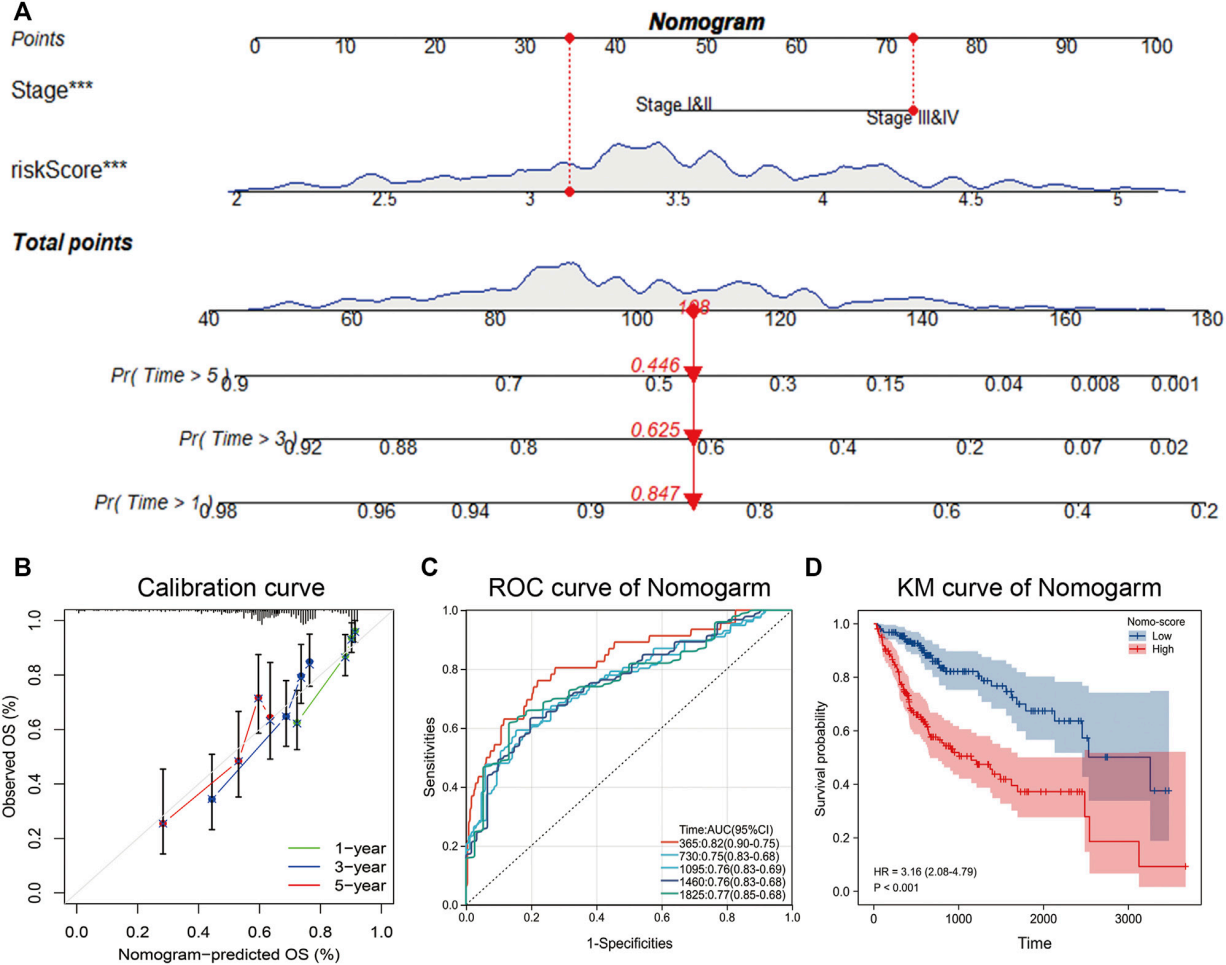
Construction of nomogram. **(A)** Acquisition of survival time and survival status of patients with hepatocellular carcinoma, and construction of nomogram by combining pathological stage and NMRS. **(B)** Calibration curve of nomogram. **(C)** Time-dependent ROC curves based on nomo-score. **(D)** Kaplan-Meier analysis based on nomo-score.

The above analysis showed that pathological stage and NMRS were independent prognostic factors, so we constructed a nomogram based on the survival status and survival time of patients with hepatocellular carcinoma (Figure 7A). By plotting the calibration curve, we found that the nomogram was close to the theoretical value in predicting 1-year, 3-year, and 5-year survival (Figure 7B). Based on the scores of nomogram, we performed time-dependent ROC analysis, and the results showed that the prediction rate of 1-year survival was 0.82, 2-year survival was 0.75, 3-year survival was 0.76, 4-year survival was 0.76, and 5-year survival was 0.77 (Figure 7C). Overall, the prediction efficiency of nomogram was within the acceptable range, and the accuracy of nomogram was high (C-index = 0.73). In addition, we also performed a prognostic analysis based on the nomo-score, and as shown in Figure 7D, the survival time of LIHC patients with high nomo-score scores was significantly shorter than that of patients with low nomo-score.

## Discussion

Metabolic aberration is an important feature of tumors. Tumor cells can satisfy their own proliferation through metabolic reprogramming (Li et al., 2021). Metabolic reprogramming can be regulated by various factors such as altered enzyme activity, differential gene expression, protein interactions, and gene mutations (Li et al., 2020). In addition, the altered metabolic environment can also affect the immune response of tumor and cause immune escape (Xia et al., 2021). Therefore, an in-depth study of the potential value of metabolism-related genes in cancer is of clinical significance.

In this study, we focused on the clinical value of nucleotide metabolism-related genes in hepatocellular carcinoma. Metabolic abnormalities in hepatocellular carcinoma exhibit a high degree of heterogeneity (Park et al., 2020), and alterations in nucleotide metabolism can be observed in different types of liver tumors (Li et al., 2021). It is well known that nucleotides (purines and pyrimidines) are the major components of human genetic material. Hepatocellular carcinoma cells use large amounts of energy and nucleotides to synthesize substances such as DNA (Yin et al., 2018). Reprogramming of nucleotide metabolic processes promotes the progression of hepatocellular carcinoma. As a result, nucleotide metabolism is a potential target for the treatment of hepatocellular carcinoma.

Based on the essential role of genes related to nucleotide metabolism in cancers, we analyzed transcriptome sequencing data of hepatocellular carcinoma using a bioinformatics approach. Potential genes were mined and a novel prognostic model was constructed. The six genes included in the model are UCK2, TXNRD1, RRM2, EMTPD2, DTYMK, and CAD. The model has good prediction efficiency in both TCGA and ICGC databases. We found that integrating the six genes into one parameter significantly improved the prediction accuracy of prognosis. This clinical parameter could well distinguish the prognosis of patients with different clinical information of hepatocellular carcinoma, especially G3 & G4 stages. In the future, the risk score calculated by the model is expected to be a powerful complement to the clinical characteristics.

Currently, the tumor immune microenvironment is a hot topic of research, and immune checkpoint inhibitors have been shown to be used for immunotherapy of tumors (Donne and Lujambio, 2022; Llovet et al., 2022). However, the prognosis of patients with hepatocellular carcinoma is hampered by the significant heterogeneity of patients and drug resistance after treatment (Cheng et al., 2020; Heinrich et al., 2021). Therefore developing and tapping targeted therapeutic strategies to improve treatment outcomes is one strategy. In this study, we compared the differences in immune checkpoint expression between high and low NMRS groups, and we also tapped more sensitive chemotherapeutic agents for patients with hepatocellular carcinoma who had a worse prognosis. Combined with better care and follow-up, this is expected to improve the survival of patients.

Numerous prognostic models for hepatocellular carcinoma have been reported previously. For example, ferroptosis-related and lipid metabolism-related prognostic model were established for prognostic analysis (Hu et al., 2020; Dai et al., 2021). However, the prognostic model constructed by nucleotide metabolism-related genes has yet to be studied in depth. Therefore, the innovation of this study is to uncover that the nucleotide metabolism-related prognostic model can be used as a complementary parameter to the clinicopathological factors. Through early psychological guidance, health education, and promotion of a healthy mindset, a positive anti-cancer process will be facilitated. Overall, the model we constructed has a high predictive accuracy compared with other reported in the literature (He et al., 2022; Zhang et al., 2022). In addition, risk assessment criteria based on NMRS could help guide clinical treatment and care of patients. This study is expected to make a potential pavement for clinical practice. Of course, there are shortcomings in this study. For example, this study requires transcriptome sequencing of a large number of hepatocellular carcinoma samples and further validation of the reliability of the results by obtaining clinical data through follow-up.

## Conclusion

Prognostic model constructed based on nucleotide metabolism-related genes have potential application in predicting the prognosis of patients with hepatocellular carcinoma. The patients with high NMRS exhibit a more malignant phenotype and have a worse prognosis due to the over-activation of cancer-promoting signaling pathways. In addition, the NMRS is expected to provide guidance for patient treatment and care as a new clinical parameter.

## Data Availability

The original contributions presented in the study are included in the article. Further inquiries can be directed to the corresponding authors.
